# The Multifaceted Roles of RCC1 in Tumorigenesis

**DOI:** 10.3389/fmolb.2020.00225

**Published:** 2020-09-15

**Authors:** Xuanqi Ren, Kai Jiang, Feng Zhang

**Affiliations:** College of Life Sciences, Shanghai Normal University, Shanghai, China

**Keywords:** RCC1, Ran, chromatin, tumorigenesis, cell cycle

## Abstract

RCC1 (regulator of chromosome condensation 1) is the only known guanine nucleotide exchange factor of Ran, a nuclear Ras-like G protein. RCC1 combines with chromatin and Ran to establish a concentration gradient of RanGTP, thereby participating in a series of cell physiological activities. In this review, we discuss the structure of RCC1 and describe how RCC1 affects the formation and function of the nuclear envelope, spindle formation, and nuclear transport. We mainly focus on the effect of RCC1 on the cell cycle during tumorigenesis and the recent research progress that has been made in relation to different tumor types.

## Ran’s Function Affects Ran-GTP Gradient

Decades of research have shown that regulator of chromosome condensation 1 (RCC1), the only known guanine nucleotide exchange factor in the nucleus for Ran (Bischoff and Ponstingl, 1995), a nuclear Ras-like G protein, directly participates in cellular processes such as nuclear envelope (NE) formation, nucleocytoplasmic transport, and spindle formation. RCC1 also regulates chromatin condensation in the late S and early M phases of the cell cycle (Dasso, 1993). The proper location of RCC1 in relation to chromatin is crucial for the functions of Ran throughout the cell division cycle (Bierbaum and Bastiaens, 2013). RanGTP gradients are generated at the nuclear pores, and this gradient across the nuclear envelop drives the nuclear cytoplasmic transport (NCT) of various cargo molecules (Bischoff and Ponstingl, 1991a,b). Ran GTPase also affects cell cycle and DNA damage response (DDR) kinetics (Thompson, 2010; Blackinton and Keene, 2014). Following disassembly of the nuclear envelope in mitotic cells, mitotic chromosomes are surrounded by diffusional RanGTP gradients, which support the assembly and function of mitotic spindles (Kalab et al., 2002, 2006; Forbes et al., 2015). RCC1 acts as a key cell cycle regulator (Ohtsubo et al., 1987) and can monitor the process of DNA synthesis RAN (Seino et al., 1991).

An increasing number of studies have found that RCC1 also plays an important role in tumors, where it mainly regulates the cell cycle process and affects tumorigenesis. RCC1 can also inhibit the occurrence of certain tumors. For example, the expression of RCC1 in gastric cancers and other tumors is significantly reduced, with different degrees of silencing occurring (Lin et al., 2015). However, in some tumors, high expression of RCC1 will also act as a pathogenic partner, promoting tumor development.

In this review, we highlight the newest findings about the RCC1’s role in the cell cycle and tumorigenesis in the context of the published data.

## Structure of RCC1

**FIGURE 1:**
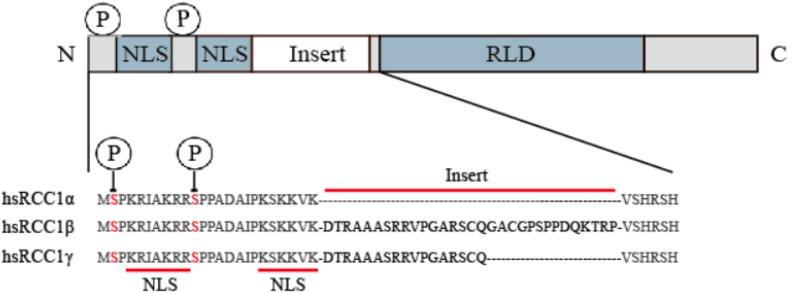
RCC1 expression of three transcript variants in humans. The RCC1 protein domain is represented linearly (not to scale), showing the alignment of the NTR of the human RCC1 protein with the insert-containing RCC1 isoform (the inserted sequence is shown in bold). Serine 2 and 11 are phosphorylation sites of NTR and are indicated by circles labeled P.

The human amino acid sequence analysis has revealed that there are three isoforms, named RCC1α, RCC1β, and RCC1γ (Hood and Clarke, 2007; Figure 1). The N-terminus contains a lysine-rich region which includes the 20-residue bipartite nuclear localization signal sequence (NLS) located on the tail of the N-terminus. The NLS regulates intracellular transport of RCC1 through the importin α/β pathway. Phosphorylation of the NLS prevents importin α/β from binding to RCC1, so that RCC1 couples the production of RanGTP to chromosome binding. N-terminal binding to chromosomal DNA requires methylation of the second serine at the N-terminal by N-terminal RCC1 methyltransferase (NRMT).

The C-terminal 7-blade β propeller domain, constituting the RCC1-like domain (RLD), has strong structural similarity with the WD40 repeat protein (Renault et al., 1998). Each blade in the beta propeller structure consists of 51–68 residue repeats and forms four antiparallel chains with loops between them. Both sides of the β-propeller structure are decorated by equivalent rings and mediate protein–protein interactions (Ruthenburg et al., 2006; Schuetz et al., 2006; Patel et al., 2008; Song and Kingston, 2008). In fact, the crystal structure indicates that one side of the RCC1 β propeller interacts with Ran via a β-hairpin extension called a β-wedge that protrudes from blade-3. RCC1 produces a RanGTP gradient around the chromosome through this binding (England et al., 2010).

## RCC1 Combines With Chromatin to Establish a Concentration Gradient of RanGTP

The cell cycle regulates RCC1 and chromatin affinity through the CDK/CyclinB1 complex. RCC1 relies on binding to chromatin, realizing the perception of chromatin state, and recruiting and converting RanGDP into RanGTP. This ability to maintain high levels of RanGTP in the nucleus, or a gradient of RanGTP concentration around the chromosome in the nuclear envelope breakdown (NEB) state, underlies the association of RCC1 with the cell cycle. Crucially, loss of RCC1 causes tsBN2 cells to condensate (Nishijima et al., 2000), indicating that the loss of RCC1 may lead to early cell cycle condensation, possibly due to the lack of RanGTP, and resulting in restricted nuclear protein output.

The structure of RCC1 bound to Ran shows that all seven blade rings on one side interact with Ran in the complex (Renault et al., 2001). Ran stabilizes the dynamic interaction of RCC1 and chromatin in living cells through the N-terminal tail of RCC1 (Hitakomate et al., 2010). Binding of RCC1 to chromatin in living cells has been studied by fluorescence redistribution (Beaudouin et al., 2006) and found to occur via the N-terminal region (NTR) of RCC1, via residues 21–25 (Seino et al., 1992), and one of the loop regions connecting the β-sheets (Bierbaum and Bastiaens, 2013). Bichromatic fluorescence correlation spectrometric measurements have shown that Ran interacts primarily with the stationary portion of RCC1, which points to catalytic sites on chromatin, and that chromatin interaction with RCC1 is more stable during metaphase than during interphase (Bierbaum and Bastiaens, 2013). It is possible that histones interact with RCC1 on the other side through the exposed spherical regions of the H2A/H2B surface (Nemergut et al., 2001; Hao and Macara, 2008; England et al., 2010). The localization of RCC1 to chromatin is critically dependent on the flexible NTR (Moore et al., 2002) which is likely to extend beyond the core structure. Chromatin interaction with RCC1 is transient (Beaudouin et al., 2006), with the residence time of RCC1 on chromatin an important kinetic parameter of the guanine nucleotide exchange reaction. The exchange response effectively binds to chromatin through the affinity of the RanGTP complex, allowing local Ran activation (Bierbaum and Bastiaens, 2013). Study of a D182A mutant found that reduced affinity between this mutant and chromatin disrupted the interaction with Ran (Azuma et al., 1999; Hutchins et al., 2004).

Because of this potential correlation between RCC1 binding to chromatin and RCC1’s Ran guanosine exchange function, multiple epigenetic modifications to the N-terminal domain of RCC1 may also influence the distribution of the RanGTP gradient. The N-terminal α-methylation of RCC1 by NRMT is important for stabilizing chromatin association and normal mitosis of cells, and RCC1 is excluded from chromosome when N-terminal tail methylation is removed RCC1 (Chen et al., 2007; Tooley et al., 2010). However, it is controversial whether RCC1 phosphorylation also affects chromatin affinity. Affinity of RCC1 for the chromosome may rely on its phosphorylation status (Hood and Clarke, 2007). The N-terminal tail of RCC1 is phosphorylated during mitosis, which inhibits binding to importin α/β (Li and Zheng, 2004). According to Hutchins et al. (2004) the phosphorylation of RCC1 is also important in allowing dynamic binding to chromatin during mitosis. Li and Zheng (2004) came to the same conclusion through the loss of fluorescence in photobleaching experiments, finding that phosphorylation leads to more stable binding to chromatin. However, Bierbaum and Bastiaens (2013), studying the dynamics of diffusion and binding of RCC1 and chromatin, found no evidence of chromatin binding regulation by N-terminal serine residue phosphorylation during mitosis.

## RCC1 Regulates Nuclear Envelope Formation, Spindle Formation and Nuclear Transport

**FIGURE 2:**
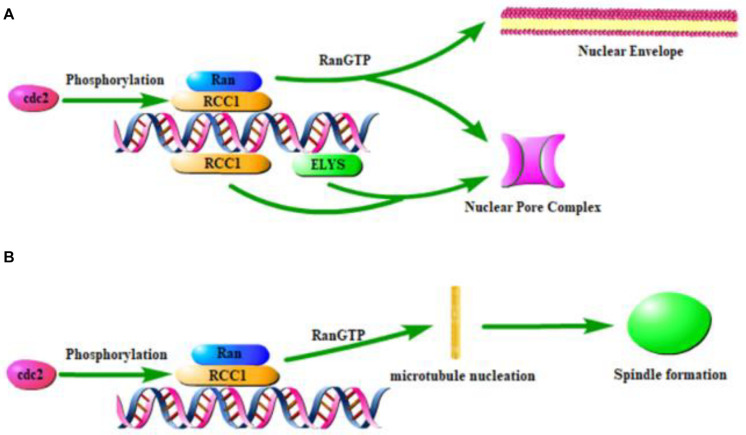
Effect of RCC1 on nuclear coating formation and spindle formation. **(A)** Model of RCC1 forming nuclear envelope and pores through chromatin. **(B)** Model of RCC1 forming spindle through chromatin.

The binding of RCC1 to chromatin is critical for nuclear envelope formation, spindle formation, and nucleocytoplasmic transport. These functions require RCC1 to combine with the nucleosomes to establish RanGTP gradients. At the end of mitosis, a new nuclear envelope (NE) is formed around chromatin, nuclear pore complexes (NPCs) are assembled in the envelope, and nuclear barrier function and nucleocytoplasmic transport are reestablished. The docking of RCC1 with H2A/H2B establishes the RanGTP gradient necessary for nuclear envelope assembly (Nemergut, 2001). Nucleosomes, but not DNA alone, mediate the chromosomal regulation of NE and NPC formation. This process first requires the generation of RanGTP by RCC1 recruited to nucleosomes (Zierhut and Funabiki, 2015). Then small GTPase Ran regulates NE/NPC assembly (Zhang et al., 2002; Horiike et al., 2009). Ran is activated by the chromatin-bound form of RCC1 (Redondo-Muñoz et al., 2015) and establishes a RanGTP gradient. NE/NPC assembly is therefore regulated by mechanisms that control RCC1 binding to chromatin (Figure 2A). This suggests that chromatin-associated RCC1 locally promotes NPC formation. Studies have shown that phosphoinositide 3-kinase β (PI3Kβ) regulates the localization of RCC1 on chromatin and subsequently the activation of Ran to exert regulation of the NE (Redondo-Muñoz et al., 2015). Localized chromatin-bound RCC1 promotes NPC formation inefficiently, which suggested that there may be a Ran independent mechanism that promotes NPC formation by nucleosomes. The nucleoporin ELYS (also known as MEL-28) can combine RCC1 with DNA and bypass the need for nucleosomes in the formation of NPC in a cooperative manner. Nucleosomes play a direct structural role in NPC recruitment by combining ELYS and RCC1 (Zierhut et al., 2014).

The location of RCC1 on the chromosome has been shown to be critical for the assembly of chromatin and RCC1-regulated spindles (Figure 2B), which requires the generation of a RanGTP gradient (Clarke and Zhang, 2008; Halpin et al., 2011; Funabiki et al., 2018; Yau et al., 2020). During mitosis in mammalian cells, GTP-bound Ran is concentrated near mitotic chromatin, while GDP-bound Ran is more abundant distal to chromosomes. This pattern spatially controls spindle formation because RanGTP locally releases spindle assembly factors (Zierhut and Funabiki, 2015). Simultaneously, local enrichment of RCC1 can be used as a factor that triggers microtubule nucleation and subsequent spindle assembly (Moore et al., 2002). During mitosis, spindle assembly in cells without centrosomes is ensured by chromosome-induced microtubule aggregation.

Phosphorylation and methylation of RCC1 also play key roles in proper mitotic spindle assembly. Notably, methylation of the RCC1 N-terminal serine residue is necessary for proper mitotic spindle assembly, which increases the affinity for chromatin (Chen et al., 2007), while phosphorylation of serines, e.g., serine 11 in humans, located in or near the NLS of RCC1 by Cdc2 kinase is necessary for the generation of RanGTP on mitotic chromosomes in mammalian cells (Li and Zheng, 2004; Zierhut and Funabiki, 2015).

The biological function of RCC1 nuclear transport is to generate a RanGTP gradient through the nuclear pore, which is then used to drive various cargo molecules to overcome their concentration gradients for transportation (Kahana and Cleveland, 1999; Dworak et al., 2019). During the early stages of apoptosis, histone modification regulates RCC1 to inhibit nuclear transport. RCC1 is immobilized on the chromosome by Mst1 phosphorylation of histone H2B at Ser 14, leading to inactivation of the nuclear transport machinery (Wong et al., 2009).

## The Role of RCC1 in Tumorigenesis

### Effect of RCC1 on the Cell Cycle

RCC1 has been shown to be a key cell cycle regulator which, in a Ran-dependent manner, monitors the process of DNA synthesis and links its completion to the occurrence of mitosis (Ohtsubo et al., 1987; Dasso, 1993). Many factors for re-entry of the cell cycle depend on nuclear cytoplasmic transport (NCT) activity regulated by RanGTP. The nuclear localization of interphase RCC1 ensures sufficient RanGTP concentration to form the driving force of NCT. For example, the realization of CyclinB1 and Gwl/Mastl kinase functions requires nuclear shuttling by importin 1 to achieve the S to G2/M phase transition (Dasso, 1993; Gavet and Pines, 2010). Twenty different NTRs interact with RanGTP such that RCC1 can run multiple NTRs simultaneously to speed up the cell cycle. In addition, there are several important large multidomain proteins that act as DNA repair regulators, and their transnuclear transport also depends on the level of RanGTP within the nucleus (Peng et al., 2013). Once spontaneous or drug-treated DNA damage occurs, the expression of RCC1 in normal cells is reduced, and the synergistic regulation of the Ran system amplifies this effect, leading to severe impairment of NCT function, which decelerates, or pauses, the cell cycle process. Alternatively, if RCC1/RanGTP is unable to respond immediately, cells carrying faulty genetic information go through the cell cycle smoothly, providing the potential for tumorigenesis.

Moreover, RCC1 is located on chromosomes in mitotic cells to maintain the gradient of RanGTP concentration around chromosomes during nuclear membrane disintegration and is involved in various genetic functions. Although RCC1 deficiency does not cause chromosome segregation defects in chicken TD40 cells, it does cause abnormalities in nuclear reconstitution, known as end-stage/G1 clover shaped abnormalities in nuclear morphology (Pemberton and Paschal, 2005). Thus, the correct location and expression of RCC1 at all stages of the cell cycle are crucial to its regulation. Of interest, Furuta et al. (2015) identified the presence of an NLS mediated nuclear localization of RCC1, through the introduction of RCC1 mutants into RCC1 deficient cells, i.e., by histone/DNA binding site of the catalytic domain, to bind chromatin and maintain binding state to the next intercellular phase by NEB state of fission (Pemberton and Paschal, 2005).

Since RCC1, ATM- and Rad3-related (ATR) kinases, and chromatin (chromosome or double-stranded DNA) all interact with each other, it is possible for RCC1 to regulate periodic monitoring points through ATR. Most likely, an ATR complex containing RCC1 is formed on chromatin after DNA damage or by inhibiting DNA replication (Abraham, 2001; Osborn et al., 2002; Furuta et al., 2015). Thus, RCC1 participates in the function of ATR cell cycle checkpoint, and this is supported by Nishitani et al.’s (2003) report of a correlation between RCC1-RAN cycles and ATR-dependent cell cycle checkpoints. If Ran is required to recruit ATR to damaged DNA or a closed DNA replication fork, RCC1 inactivation may inhibit ATR transport through the nucleoplasm. As an important functional target of ATR checkpoint, phosphorylation of Chk1 ensures DNA-induced cell cycle delay in response to unreplicated or UV-damaged DNA. Guo et al. (2000) found that the phosphorylation of Chk1 was eliminated in ATR-depleted xenopus egg extract indicating that defects in nuclear and cytoplasmic transport caused the checkpoint signal from ATR to Chk1 to be abolished (Cekan et al., 2016), which in turn weakened cell cycle arrest. Additionally, PIK-related protein kinase ATR restricts the NCT of CyclinB1-CDK1 signal by affecting CyclinB1 serine phosphorylation, ensuring the nuclear aggregation of CyclinB1 before NEB (Gavet and Pines, 2010). ATR functions as an S/G2 phase monitor during the cell cycle, responsible for preventing damaged DNA replication and inhibiting cell entry into mitosis before genome replication is complete. In this state, the reduction or inactivation of RCC1 destroys the ATR active complex composed of ATR, RCC1, and other proteins and its functions of DDR and cell cycle checkpoint. Excessive RCC1 will push the unrepaired or unreplicated cells into the division phase, also causing genomic instability and the possibility of tumorigenesis.

### Effect of RCC1 on Tumorigenesis

The coordination of cell cycle progression with the repair of DNA damage supports the genomic integrity of dividing cells. Current research data indicate that differences in expression and function of RCC1 may depend on the type of tumor.

#### RCC1 Gene Mutations Have the Potential of Tumor Development

As an integral part of cell cycle regulation, the genetic and epigenetic stability of RCC1 is crucial for cell cycle progression and maintenance of genomic stability. Therefore, mutations of the RCC1 gene have the potential for tumor development. In gastric tumor tissues, the results of differential methylation hybridization microarray analysis reflected the hypermethylation level of the RCC1 gene at the lesion site, mainly at the ninth CpG site, which caused RCC1 silencing (Lin et al., 2015). There are three specific transcription factor binding sequences (HSF1, TFIIB, and NF-X3) in this region. Oxidated nitro domain containing protein 1 (NOR1) is a candidate tumor suppressor gene, and HSF1 is a functional promoter of NOR1. The transcription factor TFIIB acts as a bridge between TFIID and RNA polymerase and can recognize the interaction of TFIIB recognition elements that are destroyed by DNA methylation (Evans et al., 2001; Kalab and Heald, 2008; Li et al., 2011). Clinicopathologically, the loss of RCC1 expression in gastric cancer leads directly to the development of tumor differentiation and invasion depth (Lin et al., 2015). In addition, the RCC1^∗^ C. 1067_1086del19 mutation found in Tunisian familial breast cancer patients also indicates that RCC1 mutations have carcinogenic potential (Riahi et al., 2018).

#### RCC1 Promotes Tumor Progression as a Pathogenic Partner

For cancers not caused by RCC1 mutations, RCC1 appears to respond to tumor cycle progression through increased expression. For example, RCC1 expression is higher in clinical cancers, such as lung adenocarcinoma, than that of normal tissues (Hsu et al., 2016). In clinical and basic studies, RCC1 is more commonly involved in tumor development and progression in this manner than direct RCC1 mutations or RCC1 gene silencing. A typical example is in cervical cancer, as based on microarray gene expression profiles, where RCC1 overexpression has only been observed in the FIGO Stage III (Thomas et al., 2013). Similarly, in genome-wide transcriptional analysis of carboplatin sensitive/tolerant ovarian cancer cells, RCC1 expression was higher in resistant cells at 2 h after carboplatin exposure, rather than being sustained throughout the entire process (Peters et al., 2005). In a study of human papillomavirus-related cervical cancer, transcription factor c-Jun directly upregulated RCC1 transcription in HPV-E7 expressing cells (Qiao et al., 2018). Similarly, the absence of mutations in the tumor suppressor PTEN in many types of human cancers also leads to increased RCC1 expression (Qiao et al., 2018). Abundant evidence indicates that RCC1 is more consistent with the role of an intermediate effector protein in tumors, and its high expression is misled by upstream signals, thus promoting the cell cycle of cancer cells.

Therefore, in cancers that do not possess a mutated RCC1, it may be possible to induce cell cycle arrest, as well as senescence or apoptosis of cancer cells by lowering the expression of RCC1. This has been demonstrated in several studies including Zhang et al. (2011), who found that 6-bromine-5-hydroxy-4-methoxybenzaldehyde was associated with the down-regulation of RCC1 protein expression during inducing mitotic catastrophe in human hepatocellular carcinoma (HCC) cells, and Qiao et al. (2018), where RCC1 knockdown inhibited G1/S cell cycle progression and DNA synthesis of HPV-E7 expressing cells. A previous study on lung cancer showed that Latcripin-13 domain, which contains a regulator of the RCC1 domain, can induce apoptosis and cell cycle arrest in human lung cancer (Wang et al., 2016). In addition, although the knockdown of KPNB1 in advanced stage prostate cancer did not affect the expression of total RCC1, it did effectively reduce the expression of downstream cycle regulators and phosphorylation of RCC1, eventually leading to cycle arrest (Yang et al., 2019). Although there is no direct evidence, it is most likely that phosphorylation of RCC1γ is reduced. It is very interesting that, among the three isoforms of human RCC1, RCC1γ, while less abundant than RCC1α, when phosphorylated exhibits a strong chromatin binding capacity, resulting in persistently high RanGTP concentrations around chromatin (Hood and Clarke, 2007). Therefore, the inhibition of RCC1 phosphorylation can also be considered as a decrease of RCC1 activity to some extent.

### RCC1 Has the Value of a Tumor Biomarker

The abnormal expression of RCC1 in a variety of malignant tumors suggests its potential as a cancer biomarker. Ideally, RCC1 is a Ran-dependent cell cycle regulator, and to some extent, its abnormal expression and epigenetic modification can effectively reflect the abnormal cell cycle of the patient’s suspected cancerous tissue. RCC1 may be used as a lone indicator or in conjunction with other biomarkers for screening to assess cancer risk and cancer progression for prediction and prognosis, respectively (Dancey, 2014). Therefore, even though there have been relatively few published reports, the biological function of RCC1 and its overexpression in multiple types of cancer appears to be relatively consistent (Hsu et al., 2016; Wang et al., 2016). However, due to highly specific mutated forms of RCC1 in a small number of cancer types, RCC1 could be used as a marker for diagnosis of these specific cancers, examples being the two highly specific fusion genes formed by RCC1 in testicular germ cell tumor (Hoff et al., 2016) and the RCC1 truncated mutation specifically observed in some Tunisian breast cancers (Hsu et al., 2016). In addition, based on bioinformatics analysis and literature search, RCC1 is one of the potential biomarkers for identifying primary lung adenocarcinoma (Wong et al., 2009).

In conclusion, although RCC1 is still rarely used directly in the clinical diagnosis of cancer, it is valuable to determine the reference normal range of RCC1 in various tissues.

## Discussion

In recent years, an increasing number of studies have found that RCC1 is related to cell cycle, DNA damage, and cancer.

As an important cell cycle regulator, RCC1 affects the progress of the cell cycle. When DNA is damaged, the decrease in RCC1 expression in normal cells may lead to severe damage to NCT and affect re-entry of the cell cycle (Cekan et al., 2016). The continuous accumulation of DNA damage is also a major cause of cancer. The loss of ATR-related cell cycle monitoring points increases the risk of DNA damage accumulation and increases the likelihood of tumors (Guo et al., 2000). The high expression of RCC1 in cancer cells accelerates the cell cycle and DNA repair, and, as such, tumor cells may regulate cell mitosis by increasing the expression of RCC1. RCC1 accelerates the formation of the nuclear membrane and the spindle to promote the mitotic process of cells. However, the mechanism by which RCC1 DNA damage responds to the cell cycle through the exact NCT or specific cell function requires further study.

Interestingly, RCC1 has different expression profiles and functions in different tumors. On the one hand, RCC1 expression is negatively correlated with the development of certain tumors. Targeting RCC1 can induce tumor cell apoptosis and cell cycle arrest (Hsu et al., 2016). RCC1 mutations or methylation can be key to tumor development, with this process showing the potential to inhibit tumors and regulate DNA replication (Lin et al., 2015). Already, there are nanoparticles treatments containing inhibitory peptides targeting RAN that have great potential in therapy of breast cancer (Haggag et al., 2019). On the other hand, RCC1 can also promote tumorigenesis. ERK1/2 can increase the expression of RCC1 through c-Jun, which affects the genome stability and promotes the development of tumors. ERK1/2 signaling could promote the development of osteosarcoma via regulating H2BK12ac (Xu et al., 2019). Histone interacts with RCC1 through the H2A/H2B surface area. Therefore, ERK1/2 may increase the expression of RCC1 through the c-Jun pathway to regulate H2BK12ac. Phosphorylation of RCC1 can affect its binding to chromosomes and also inhibit the proliferation of certain tumors. Its mechanism of action also has research value.

At the same time, RCC1 has different expression profiles in different tumors and shows promise as a potential biomarker. The reason for the different effects of RCC1 on different tumor types is not yet clear, but research on its role in the cell cycle, apoptosis, and genome stability has significant prospects.

## Author Contributions

XR: manuscript writing. FZ: review and modify. All authors contributed to the article and approved the submitted version.

## Conflict of Interest

The authors declare that the research was conducted in the absence of any commercial or financial relationships that could be construed as a potential conflict of interest.
